# Temporal interpolation of real-time cine images for ventricular function assessment

**DOI:** 10.1186/1532-429X-16-S1-O42

**Published:** 2014-01-16

**Authors:** Haris Saybasili, Gary R McNeal, Sven Zuehlsdorff, Michaela Schmidt, Peter Kellman, Michael O Zenge

**Affiliations:** 1 Siemens Healthcare USA, Inc., Chicago, Illinois, USA; 2 Siemens AG Healthcare Sector, Erlangen, Germany; 3 National Heart Lung and Blood Institute, National Institutes of Health, Bethesda, Maryland, USA

## Background

In uncooperative patients or in presence of arrhythmia, real-time free-breathing cine MR approaches may offer an alternative to well-established segmented data acquisition strategies [1,2]. Segmented acquisition strategies collect data throughout a number of heartbeats and typically a fixed number of cardiac phases are reconstructed for each slice representing an average RR interval. In real-time cine imaging, the number of reconstructed phases may be different for each slice due to heart rate variations. However, it is desirable to reconstruct a predefined number of cardiac phases per slice to facilitate functional analysis and processing. In this work, we present an image reconstruction approach that retrospectively interpolates real-time cine images to calculate a predefined number of cardiac phases per heartbeat and slice. Ejection fraction is compared between segmented cine images and interpolated real-time cine images in healthy volunteers.

## Methods

**Figure 1:**
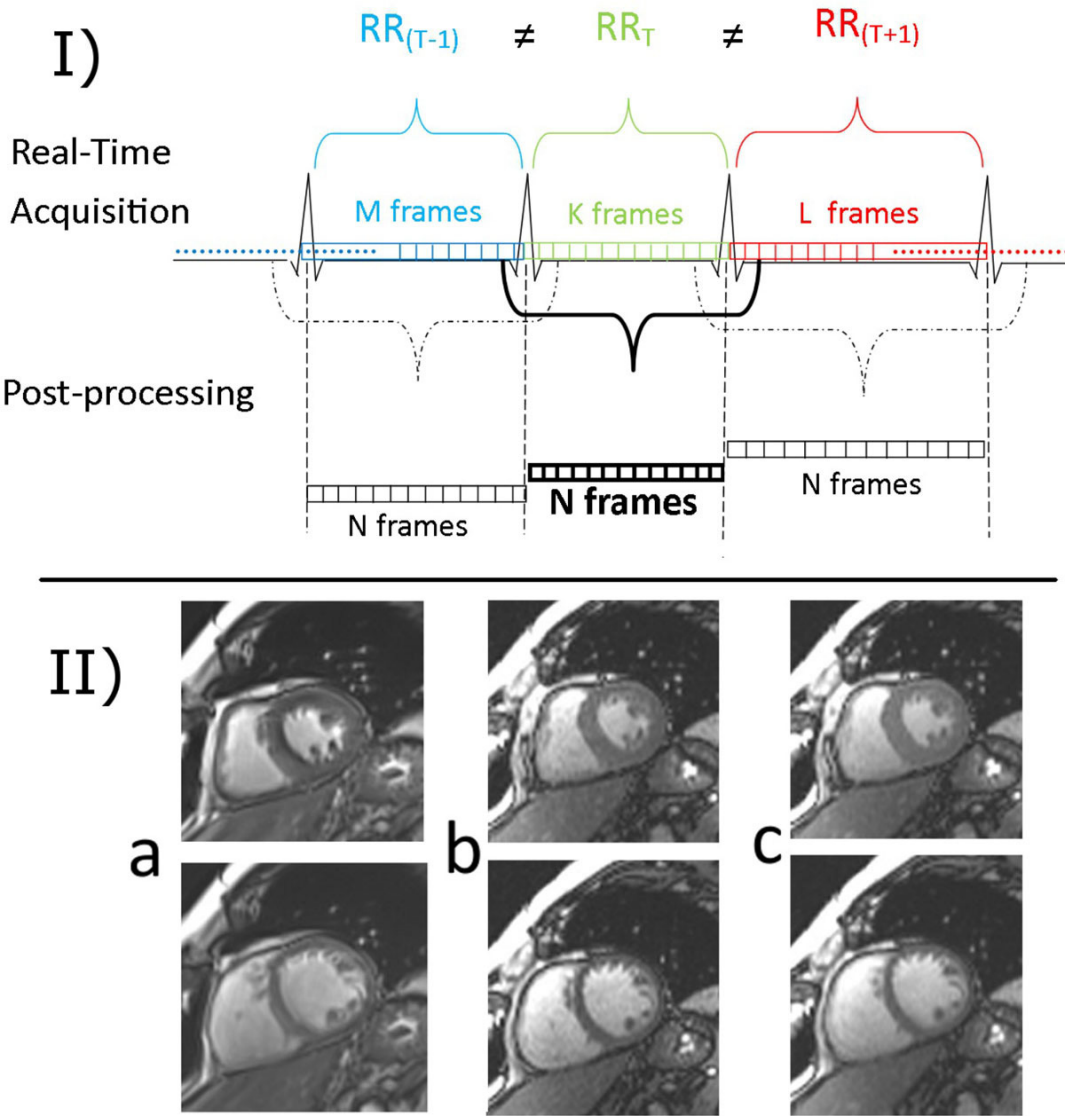
**I. Interpolation of RT cine images**. Real-time acquired images during a heartbeat are interpolated to a fixed number of frames. The linear interpolation algorithm uses frames before and after the QRS peak to estimate first and last phase. II. End-systolic (upper images) and end-diastolic (bottom images) cardiac phases for a) segmented, b) non-interpolated real-time, and c) interpolated real-time images. Please note that interpolation introduces a slight blur to the images.

Segmented breath-hold and real-time free-breathing cine images were acquired in four healthy volunteers on a clinical 3T MR scanner (MAGNETOM Skyra, Siemens Healthcare, Erlangen, Germany). Acquisition parameters for each volunteer are represented in Table 1. FOVs for real-time studies were kept as small as possible to improve spatial resolution. T-PAT factor 3 was used to improve temporal resolution. The linear interpolation module was fully integrated into the reconstruction pipeline of the scanner. During imaging, heart rate was continuously monitored and real-time images were linearly interpolated to a fixed number of cardiac phases for each heartbeat and for each slice (Figure 1). 20 cardiac phases were calculated per heartbeat by interpolation during real-time acquisitions to achieve 50 ms nominal temporal resolution. Subsequently, interpolated real-time images were analyzed to evaluate LV function (syngo Argus 4D ventricular function, Siemens Healthcare, Erlangen, Germany) and compared with results based on segmented images.

**Table 1:** 

Data acquisition parameters for healthy volunteers				
	Segmented	Real-Time (RT)	RT Interpolated	
Temporal Resolution (ms)	39 to 41	78 to 114	42 to 55	
Spatial Resolution (mm)	1.5 × 1.5 × 6	2.8 × 2.3 × 6	2.8 × 2.3 × 6	
				
Comparison of ejection fraction values between segmented and interpolated real-time data sets (Seg: segmented, RT: real-time).				
	Volunteer 1 Seg./RT	Volunteer 2 Seg./RT	Volunteer 3 Seg./RT	Volunteer 4 Seg./RT
EF (%)	57/57	65/64	65/64	68/65

## Results

Interpolated real-time images were loaded to Argus and endo-cardial borders were detected prior to analysis. EF measurements (see Table 2) from interpolated real-time and segmented images were found to be in good agreement. End-systolic and end-diastolic phases for segmented, non-interpolated real-time, and interpolated real-time images are given in Figure 2. Interpolation increased the SNR, but introduced slight blurriness. The level of blurring was low and thus it did not affect the functional evaluation.

## Conclusions

In this feasibility study we presented initial evidence that interpolated real-time cine images may be used to facilitate cardiac functional analysis using well-established post-processing software. The current implemented algorithm will be extended to take arrhythmia events into account.

## Funding

The author is a full-time employee of Siemens Healthcare USA, Inc.
